# Epidemiological profile and spectrum of neglected tropical disease eumycetoma from Delhi, North India

**DOI:** 10.1017/S0950268819001766

**Published:** 2019-10-22

**Authors:** N. Dubey, M. R. Capoor, A. S. Hasan, A. Gupta, V. Ramesh, S. Sharma, A. Singh, S. M. Rudramurthy, A. Chakrabarti

**Affiliations:** 1 Department of Microbiology, Vardhman Mahavir Medical College & Safdarjung Hospital, New Delhi, India; 2 Department of Dermatology, Vardhman Mahavir Medical College & Safdarjung Hospital, New Delhi, India; 3 Department of Pathology, Vardhman Mahavir Medical College & Safdarjung Hospital, New Delhi, India; 4 Department of Medical Microbiolgy, Postgraduate Institute of Medical Education and Research, Chandigarh, India

**Keywords:** Epidemiological profile, eumycetoma, neglected tropical disease, spectrum

## Abstract

Mycetoma is a chronic granulomatous, suppurative and progressive inflammatory disease that usually involves the subcutaneous tissue and bones after traumatic inoculation of the causative organism. In India, actinomycotic mycetoma is prevalent in south India, south-east Rajasthan and Chandigarh, while eumycetoma, which constitutes one third of the total cases, is mainly reported from north India and central Rajasthan. The objective was to determine the epidemiological profile and spectrum of eumycetoma from a tertiary care hospital in Delhi, North India. Thirty cases of eumycetoma were diagnosed by conventional methods of direct microscopy, culture and species-specific sequencing as per standard protocol. The spectrum of fungal pathogens included *Exophiala jeanselmei*, *Madurella mycetomatis*, *Fusarium solani*, *Sarocladium kiliense*, *Acremonium blochii*, *Aspergillus nidulans*, *Fusarium incarnatum, Scedosporium apiospermum* complex, *Curvularia lunata* and *Medicopsis romeroi*. Eumycetoma can be treated with antifungal therapy and needs to be combined with surgery. It has good prognosis if it is timely diagnosed and the correct species identified by culture for targeted therapy of these patients. Black moulds required prolonged therapy. Its low reporting and lack of familiarity may predispose patients to misdiagnosis and consequently delayed treatment. Hence health education and awareness campaign on the national and international level in the mycetoma belt is crucial.

## Introduction

Mycetomas are chronic subcutaneous infections caused by fungi or actinomycetes, known as eumycetomas or actinomycetomas respectively [1, 2].

Mycetoma has a worldwide distribution, mainly confined to tropical regions in the area between the latitudes of 15°S and 30°N known as the ‘Mycetoma belt’ [3]. India and Sudan have higher than average prevalence of the infection. Regional distribution of mycetoma includes Sri Lanka, India, Pakistan, Sudan, Senegal, Somalia, Mexico and South America [3]. In India, Rajasthan (North-West India) and South India are mostly affected. Eumycetoma constitutes one-third of the total cases mainly reported from Uttar Pradesh (North India) and Central Rajasthan, while actinomycotic mycetoma is usually present in South India, South-East Rajasthan and Chandigarh [4, 5].

**Fig. 1.**
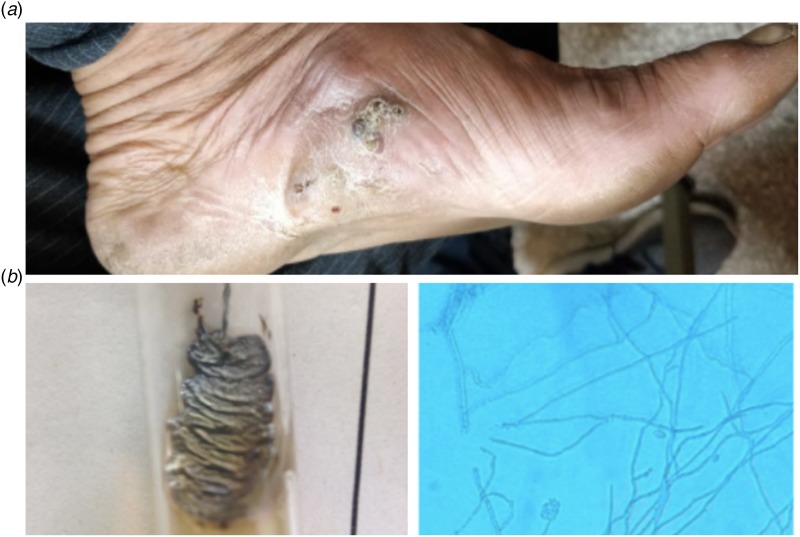
(a) Granulomatous lesion with sinus tract formation and discharging black grains, tumefaction and (b) Colony morphology and microscopic picture of *E. jeanselmei*.

The organisms are chronic infections which are traumatically implanted into the subcutaneous tissue and deeper structures like fascia, joints and bones from the natural environment. They cause a subcutaneous infection characterised by progressive granulomatous lesions, with or without sinus tract formation with discharging grains, tumefaction and spreading into the adjacent tissue, bone, fascia and ligaments (Fig. 1) [6–9]. The main sites affected are lower limb and upper limb. Uncommon sites include trunks, buttocks, eyelids, lacrimal glands, paranasal sinuses, nails, mandible, scalp, neck, perineum and testes [6].

Black grain eumycetoma includes *Madurella mycetomatis, Trematosphaeria grisea*, *Exophiala jeanselmei* and *Curvularia geniculata*. White grain eumycetoma includes *Scedosporium apiospermum* complex, *Aspergillus nidulans*, *Acremonium falciforme and Fusarium species* [10].

Prompt diagnosis and rapid treatment is required in the cases of eumycetoma. It is a chronic disease and the relapse rate is very high due to this it has become one of the most neglected disease and important public health problem in Africa and south Asia [11]. Anti-fungal susceptibility testing is still in its evolving stage. That is why correct identification of the species is of paramount importance [1, 2, 10].

The current study was performed to know the epidemiological profile and spectrum of eumycetoma in Delhi, North India.

## Methods

Thirty patients with eumycetoma seen in the last 13 years (January 2004–June 2018) were evaluated retrospectively at the Department of Microbiology, VMMC and Safdarjung Hospital. All the cases of actinomycetoma were excluded from the present study.

A diagnosis was made clinically on the basis of the classical triad of tumefaction, discharging sinuses and the presence of grains in these sinuses (Fig. 1a). A total of 59 samples were received.

The age, gender, occupation, site of involvement, duration of disease and underlying bony involvement detected by X-ray examination of patients were recorded. The size, shape, colour and consistency of granules were examined macroscopically. Direct examination of the granules was done by washing in normal saline followed by making a 10% KOH mount. The size of hyphae, septations and pigment formation in the hyphal walls was examined under a microscope. In cases of non-sporulating mould, slide culture was put up. The eumycotic grains appeared as 2–6 µm wide hyphae, have large, globose swollen cells (up to 15 µm) at margins. Gram's stain preparation was made by crushing the grain or tissue sample between two slides, heat fixing and staining. Gram's staining and Kinyoun's modified acid fast staining using 1% sulphuric acid were done [12, 13].

When no growth was obtained, the etiologic description was restricted to grain colour and/or actinomycotic/eumycotic aetiology [14]. The organisms were identified on the basis of their cultural characteristics and morphology and conidiation pattern on lacto phenol cotton blue (LPCB) mount. On LPCB mount the varied appearances were identified as follows:

1. Sub spherical cells with annellidic butt producing long chains of conidia. The conidiogenous cells of hyphae were seen as intercalary or rocket shaped and these were identified as *E. jeanselmei* (Fig. 1b).
2. *M. mycetomatis* showed small conidia that are ovoid to globose, on corn meal agar pointed conidiophores bearing conidia at the tips of flask-shaped phialide was seen.
3. *Fusarium solani* appeared as single or grouped conidiophores, conidia were seen in conidial balls with hyaline transverse septae.
4. *Sarocladium kiliense* had slimy colonies with pinkish appearance on SDA, microscopically hyaline hyphae with scanty sporulation were seen.
5. *A. nidulans* had globose conidia surrounded by mass of hulle cells.
6. *Fusarium incarnatum* showed hyaline hyphae with clustered and chain of verrucose chlamydospores and coiled sterile hyphae and characteristic curved slender septate macroconidia. Sporodochia were produced on a piece of carnation leaf.
7. *S. apiospermum* complex had broad hyphae of up to 2–5 µm width, cleistothecia formation was seen, conidia were single celled pale brown and oval in shape (Fig. 1c).
8. *Curvularia lunata* had simple brown coloured conidiophores bent at points showing sympodial geniculate growth, conidia had 3–4 septae at third cell from base.
9. *Medicopsis romeroi:* Lacto phenol cotton blue mount of the colonies revealed brown septate hyphae. The microslide culture on oat meal agar which revealed a grey black mould after 5 weeks. Lacto phenol cotton blue mount showed brown septate mycelium with brown-black pycnidia.
10. *Lasiodiplodia theobromae:* Lactophenol cotton blue mount showed brown septate mycelium with brown-black pycnidia, paraphyses and immature conidia (whitish) with thin walls, and mature conidia (dark brown) with septa and thick walls.
11. *Alternaria alternata:* simple brown coloured conidiophores with muriform conidia.

Few nonsporulating isolates and rare fungal isolates were further sent for reconfirmation and sequencing of the internal transcribed spacer (ITS) ITS1, ITS2, ITS4 and D1, D2 sequencing regions of ribosomal DNA (rDNA) at National Culture Collection for Pathogenic Fungi (NCCPF), Post Graduate Institute of Medical Research and Education (PGIMER), Chandigarh, India. Out of these *M. romeroi* (Accession No: MK 955353, NCCPF 830008) *and E. jeanselmei* (Accession No: MK955352, NCCPF 106021) were sequenced and were then alloted NCCPF (National Culture Collection for Pathogenic Fungi) collection numbers. Strains of *F. incarnatum* (1), *F. solani* (2), *S. kiliense* (1), *E. jeanselmei* (1), *Acremonium species* (1), *S. apiospermum complex* (1), *L. theobromae* (1), *Aspergillus flavus* (1), *A. alternata* (1) and *Aspergillus terreus* (1) were reconfirmed in reference centre on culture and then given NCCPF (National Culture Collection for Pathogenic Fungi) collection numbers for future reference. Rest strains could not be revived (Refer to Table 1).

**Table 1.** Distribution of the patients according to etiological agent, laboratory diagnosis, treatment and outcome of therapy

S. no	Causative organisms	Total cases	Age/gender	Colour of grain	Microscopy	Laboratory diagnosis	Therapy	Response to therapy
1.	*A. flavus*	6	Range 25–35 years/M 28/F 23/F	White	Conidial heads radiate, later form loose columns biseriate, some heads with phialides borne directly on the vesicle (uniseriate). Hyaline conidiophore stipes. Conidia are globose to subglobose (3–6 µm in diameter).	Microscopy and culture ILK 588	Amphotericin B Itraconazole	All cured
2.	*A. nidulans*	4	17/M 23/M 37/M 42/M	White	Colonies are typically plain green in colour with dark red-brown cleistothecia developing within and upon the conidial layer. Conidial heads are short, columnar and biseriate. Short brownish conidiophore stipes. Conidia are globose (3–3.5 µm in diameter) and rough-walled.	Microscopy and culture	Amphotericin B Itraconazole	All cured
3.	*A. terreus*	1	20/M	White	Colonies are typically cinnamon brown in colour	Microscopy and culture ILK 782 NCCPF 860032	Amphotericin B Itraconazole	Cured
4.	*F. solani*	4	21/M 29/M 19/F 24/F	White	Rapidly growing colonies, aerial mycelium white to cream. Macroconidia are formed from short multiple branched conidiophores. They are three to five-septate, fusiform, cylindrical. Microconidia are cylindrical to oval, one to two-celled and formed from long lateral phialides.	Microscopy and culture 2 strains sent for reference centre: NCCPF 58A0064, (ILK-518) NCCPF 58A0082, (ILK-780)	Amphotericin B Itraconazole	One patient complained of recurrence rest 3 fully cured.
5.	*S. kiliense*	4	22/M 27/M 29/M 43/M	White	White-yellow, 20 mm, irregular shape, with fragmented hyphae Hyaline hyphae, simple conidiophore and apical conidia clustered into a circle kept together by mucilaginous substances	Microscopy and culture NCCPF No.: 530034	Amphotericin B Itraconazole	Two complained of recurrence, rest 2 fully cured.
6.	*C. lunata*	2	26/M 29/M	Black	Colonies black, downy. Conidiophores erect, unbranched, septate. Conidia smooth-walled, olivaceous brown, 3-septate, the subterminal cell swollen and distinctly larger than the remaining cells.	Microscopy and culture	Amphotericin B Itraconazole	All cured
7.	*E. jeanselmei*	1	28/M	Black	Mature conidiogenous cells rocket-shaped, slightly darker than the supporting hyphae, with regular tapering annellated zones.	Microscopy and culture NCCPF-106021 (ILK-692)^a^ Sequencing Accession No: MK 955352	Itraconazole	Cured
8.	*M. mycetomatis*	1	33/F	Black	Yellow or brown, ridged surfaces Moniliform, dematiaceous hyphae and chlamydospores, 25 µm in diameter; simple and branching conidiophores.	Colony morphology Microscopy	Ketoconazole	Recurrence followed by amputation
9.	*Acremonium kiliense*	1	27/M	White	Colonies slow growing, moist at first, suede-like later. Hyphae are fine and hyaline and produce awl-shaped erect phialides with inconspicuous collarettes. Conidia are usually one-celled, hyaline or rarely pigmented, globose to cylindrical.	Colony morphology Microscopy NCCPF 530034	Amphotericin B Itraconazole	Recurrence
10.	*Acremonium* spp.	1	20/M	White	Moist, white colonies. Hyphae are fine and hyaline and produce awl-shaped erect phialides with inconspicuous collarettes. Conidia are usually one-celled.	Colony morphology Microscopy NCCPF 530058	Amphotericin B Itraconazole	Recurrence
11.	*Acremonium blockii*	1	21/M	White	Colonies slow growing, moist at first, suede-like later. Hyphae are fine and hyaline and produce awl-shaped erect phialides with inconspicuous collarettes. Conidia are usually one-celled, hyaline or rarely pigmented, globose to cylindrical.	Colony morphology Microscopy ILK 292	Amphotericin B Itraconazole	Treated
11.	*F. incarnatum*	1	19/M	White	Conidiophores scattered in the aerial mycelium, loosely branched; polyblastic conidiogenous cells abundant. Sporodochial macroconidia slightly curved, with foot-cell, three to seven-septate. Conidia on aerial conidiophores (blastoconidia) usually borne singly on scattered denticles. Microconidia sparse or absent.	Colony morphology Microscopy NCCPF 580079 (ILK-767)	Itraconazole	Recurrence
12.	*S. apiospermum/Pseudaallescheria boydii*	1	31/M	White	Fast filamentous growth, cottony dark-grey mycelium Isolated annelloconidia formed at the apex of annellospores. Pyriform aleuriospores distributed in the apices of simple or branched conidiospores. Strands of conidiospores form coremia.	Microscopy and culture ILK-563	Amphotericin B Voriconazole	Recurrence
13.	*M. romeroi*	1	24/M	Black	Brown septate hyphae. The microslide culture on oat meal agar which revealed a grey black mould after 5 weeks. Lacto phenol cotton blue mount showed brown septate mycelium with brown-black pycnidia Sequencing	Microscopy and Culture NCCPF 830009 (ILK −684)^a^ Accession No: MK 955353	Amphotericin B	Recurrence
14.	*L. theobromae*	1	34/M	Black	Brown septate mycelium with brown-black pycnidia, paraphyses, immature conidia (whitish) with thin walls, and mature conidia (dark brown) with septa and thick walls.	Colony morphology Microscopy NCCPF 670005 (ILK961)	Amphotericin B Itraconazole	Recurrence
15.	*P. chrysosporium*	1	28/M	Black	Simple-septate generative hyphae; single or multiple clamps may be present in the subiculum. The basidia (spore-bearing cells) are club-shaped and smooth. Spores of the genus are thin-walled, hyaline, and have a cylindrical to ellipsoidal shape	Colony morphology Microscopy	Amphotericin B	Recurrence
16.	*A. alternata*	1	30/F	Black	Brown coloured hyphae with muriform conidia	Colony morphology Microscopy NCCPF 650028 (ILK 778)	Amphotericin B	Cured

M, male; F, female; NCCPF, National Culture Collection for Pathogenic Fungi.

a Collection Numbers.

All patients with eumycetomas were managed with surgical debridement and either of oral antifungal drugs such as ketoconazole, voriconazole, itraconazole and intravenous amphotericin B and periodic surgical debridement.

## Results

A total of 59 skin tissue samples were received from the Department of Surgery and Department of Dermatology, out of which 30 cases of eumycetoma, three cases of actinomycetoma were diagnosed and 26 cases showed no growth. Thirty cases of eumycetoma were analysed. The age group included young adults (15–45 years), males were affected more than females. The microscopic examination of thirty samples (10% KOH) revealed fungal elements and fungal hyphae and yielded fungal growth. Thirty cases yielded eumycotic aetiology, three cases yielded actinomycotic aetiology and rest 26 samples were showing no growth on prolonged incubation. In the present study, only the species of eumycetoma are taken into consideration. The age group included young adults (15–45 years), males (24) were affected more than females (6). All of them were agricultural workers or labourers involved in construction work. The patients were from Rajasthan (12), Bihar (7), Haryana, (4), Uttar Pradesh (4), Madhya Pradesh (1), West Bengal (1) and Delhi (1). The spectrum of Mycetoma obtained was *A. flavus* (6), *A. nidulans* (4), *F. solani* (4), *S. kiliense* (4), *C. lunata* (3), *E. jeanselmei* (1), *M. mycetomatis* (1), *Acremonium blochii* (1), *F. incarnatum* (1), *S. apiospermum complex* (1), *M. romeroi* (1), *L. theobromae* (1), *A. alternata* (1), *A. terreus* (1) and *Phanerochaete chrysosporium*.

A total of 22 isolates of white grain mycetoma and eight isolates of black grain mycetoma were diagnosed.

Table 1 shows distribution of the patients according to etiological agent, laboratory diagnosis, treatment and outcome of therapy. Grains of eumycetoma cases were characterised by the presence of parallel running hyphae with or without chlamydospores and were better delineated on intense positivity in periodic acid Schiff (PAS) stain. The grains of *M. mycetomatis* were larger and had foreign body type of giant cell reaction around them.

In the histology section of the grain of *M. mycetomatis*, the filamentous grain consists of brown septate and branched hyphae that were slightly more swollen towards the periphery. In the black grains of *Falciformispora senegalensis* and *T. grisea* the centre was non-pigmented and cement was absent, whereas at the peripheries the grains were dark coloured and brown cement was present. It was difficult to distinguish between *F. senegalensis* and *T. grisea* based on histology alone.

The criteria for cure includes disappearance of the subcutaneous mass, healing of the sinuses and the skin return to normal, the bone regains its normal radiological appearance with remodelling, the absence of hyper reflective echoes and cavities on ultrasonic examination and no grains seen in fine needle aspiration.

The wound was surgically debrided and antifungal therapy was initiated. Eleven patients in the present study showed response to treatment by antifungal therapy. The antifungal therapy ranged from amphotericin B, ketoconazole, itraconazole and voriconazole. We observed a high drop-out rate in the patients. Ten out of 30 patients (33.33%) had multiple surgical excisions and had recurrence. Out of the 30 cases, one patient's lower limb had to be amputated and two patients could not be followed up. This patient developed a nodule in the anterior aspect of shin after the trauma and had tibial involvement on X-ray after the disease progression. *M. mycetomatis* was isolated in this patient. However, despite antifungal therapy, the patient underwent limb amputation.

## Discussion

Mycetoma is defined as a chronic subcutaneous granulomatous reaction caused by traumatic implantation of either true fungi or aerobic bacteria present in the soil [1]. It is also known as ‘Madura foot’ because it was first described in Madurai (South India) in 1842 [3]. Presently, more than 70 species are proven as agents of mycetoma [15].

Mycetoma predominantly affects men as compared to women especially in rural areas, and it is mostly seen in patients who work barefoot on land such as farmers and daily labourers [1]. In the present study, 24 of 30 patients were men and this is in accordance with previous reports from the Sudan [1]. Males were mostly affected in our series and this is in agreement with preceding reports from the Sudan and globally, however, the sex ratio reported in this series is smaller [15–18]. The explanation for this is unclear and suggested that sex hormones might play a role in this predominance [10]. All of them were farmers and labourers in our case series. This is an important finding as the nature of their work puts them in direct contact with the soil on a daily basis and it has been postulated that the soil harbours the causative organisms and these patients are constantly exposed to minor injuries which facilitate the traumatic subcutaneous inoculation of the organisms [1]. The patients were mainly from north Indian states closer to Delhi like Rajasthan, Uttar Pradesh, Haryana, Bihar, Madhya Pradesh. In India, eumycetoma constitutes one-third of the total cases mainly reported from Uttar Pradesh and Central Rajasthan in North India and few states in South India [4, 5].

The majority of the reported patients were young adults with a mean age of 25 ± 15 years and this is a typical age in mycetoma patients [3]. In endemic regions, any age group can be affected, although it mostly affects young individuals of age group 20–40 years. The young adults were most frequent affected cohort in the present study which is in agreement with other series [15–18]. In developing country, the young adults are often the working force and therefore mycetoma in these patients leads to serious socio-economic consequences [18]. Risk factors include low-socioeconomic status, insufficient nutrition and poor hygiene [6]. The entry of the causative agent into the subcutaneous tissue is through abrasion of the skin or through traumatic implantation [6]. The most common site of involvement was the lower extremities especially the foot (22 of 30, 73.3%) [1]. The extra-pedal sites of involvement in this study were hands and trunk. The mean disease duration at presentation among the affected study population is quite long. This may be explained by the painless nature of the disease, the lack of health education, low socio-economic status of the affected patients and lack of medical and health facilities in the endemic regions [19]. The clinical presentation of patients in this series was typical and in agreement with other reports [3]. It started gradually at the subcutaneous tissue and progressed to affect the deep structures. It was painless in the majority of patients and that may be an important contributory factor for the late presentation in most patients.

The study showed that 10 out of 30 patients (33.33%) had multiple surgical excisions and recurrence and had surgery. The treatment dropouts were high and they were likely related to delayed clinical responses and the prolonged treatment times. Recurrence was also frequent [20] and prevailed in patients that had undergone surgery [21]. The reasons are unknown, but may be likely due to the existence of undiagnosed subclinical lesions fungal defence mechanisms against antifungal drugs or incomplete surgical procedures. It is a well-known fact that incomplete surgical excision performed under local anaesthesia is the major factor leading in recurrence [10]. At presentation majority of the patients had massive ulcers which is caused by their late presentation, and also eumycetoma are known to be aggressive and can invade the deep structures and bone at an early disease stage. Sequence based identification of black granule mycetoma agents facilitated identification because many black grain producing fungi do not sporulate or require prolonged incubation to do so. Species specific sequences allowed differentiation of *M. mycetomatis* and other eumycetoma agents: *C. lunata, F. senegalensis, T. grisea* and *M. romeroi* [3] as was also done of nonsporulating and rare fungi in this study [3].

Indian studies by Desai *et al*. highlight the most common site as lower limb with majority of cases of actinomycetoma diagnosed [8]. The other Indian study by Mathur *et al*., diagnosed most of the cases as eumycotic mycetoma with lower extremities affecting in most of the patients [22]. This is in accordance with the present study. Maiti *et al*., studied between 1981 and 2000, 264 cases of mycetoma were diagnosed clinically and microbiologically at Calcutta School of Tropical Medicine, Kolkata, East India [23]. Retrospective analysis of the records revealed that the ratio of actinomycetomas and eumycetomas was 197 : 67; the male to female ratio was 183 : 81. Ninety-four cases occurred in the 1980s and 170 in 1990s, with significantly more infections of *Actinomadura* spp. (*P* < 0.01) and fewer with *Nocardia caviae* (*P* < 0.01) during the last decade. Pricking was the most common injury associated with eumycetomas (*P* < 0.01). A total of 196 infections were in exposed body parts and 68 in covered areas. The localisation of mycetomas differed significantly (*P* < 0.01) according to sex, incidence of actinomycetomas or eumycetomas, and obvious history of trauma. Exposed area cases were more common among agricultural workers (*P* < 0.01), while covered area mycetomas were almost always actinomycetomas with a remarkably lower incidence of *N. caviae*, *A. madurae and Madurella grisea* infections. The peak age of onset was between 16 and 25 years. In the present study also, the mean age for presentation is in accordance with other Indian studies [4, 5, 8, 9].

Dieng *et al*., in their analysis of 130 cases of mycetoma from Senegal, had 76 cases of actinomycetoma and 54 cases of eumycetomas [24]. The commonest isolates among actinomycetomas were *Actinomadura pelletieri and Actinomadura madurae* whereas among eumycetomas were *M. mycetomatis* and *S. apiospermum* complex. The predominant etiologic agents in Mexico differed from those in Sudan. *Nocardia brasiliensis* (86%) and *Actinomadura madurae* (10%) were the most common etiologic agent in Mexico [25].

Table 2 shows the comparative data between this study and the other studies.

**Table 2.** Comparative data between this study and the other Indian studies

Author	Year	Place of study	Total	AM	EM	Most common site/sites	Most common species isolated
Desai *et al*. [8]	1970	Bombay, India	40	40	Nil	Foot	*Nocardia*
Reddy *et al*. [9]	1972	Vishakhapatnam, India	50	36	14	Foot	*Streptomyces madurae, M. mycetomatis*
Venugopal TV *et al*.	1977	Madras	90	61	29	Lower extremities	*Nocardia spp, A. pelletieri*
Singh *et al*. [4]	1979	Rajasthan, India	262	39	223	Extremities, perineum	*M. mycetomatis, Streptomyces madurae*
Talwar *et al*.	1979	North India	60	48	12	Foot/lower extremities	*A. pelletieri, Actinomadura madurae, Phialophora jeanselmei*
Mathur *et al*. [15]	2008	Jodhpur, Rajasthan	73	25	48	Lower extremities	*M. mycetomatis*
Somnath Padhi *et al*.	2009	Hyderabad, India	13	8	5	Foot/lower extremities	*M. mycetomatis, Actinomadura madurae*
This study	2017	New Delhi, India	33	3	30	Foot/lower extremities	*A. flavus (7), A. nidulans (4), F. solani (4), S. kiliense (3), C. lunata (3)*

The present study showed poor treatment outcome, only 20 patients were cured and this is in line with previous reports [24]. In the present study, amphotericin B and itraconazole combination was given for *A. flavus*, *A. nidulans*, *F. solani*, *S. kiliense*, *C. lunata*, *A. blochii* and *L. theobromae*. The isolates of *E. jeanselmei* and *F. incarnatum* were treated with itraconazole alone, *M. mycetomatis* was treated with ketoconazole alone. The cases of *M. romeroi* and *Phanerochaete chrysosporium* were treated with amphotericin B alone. Eumycetoma has no acceptable treatment at present due to the presence of entangled hyphae making the blood grain barrier making the penetration of the drug very difficult [24]. Reports on medical treatment in eumycetoma are scarce and disappointing. Over the years the treatment of eumycetoma was based on personal clinical experience and on the results of sporadic case reports, rather than controlled clinical trials. Still, in many centres, massive surgical excisions or amputation of the affected part are the treatment of choice [16, 26]. Amphotericin B has been used with limited success, and it is no longer popular due to its serious toxic side effects [27]. The most popular treatment regimens nowadays for eumycetoma are ketoconazole 400–800 mg/day or itraconazole 400 mg/day for extended periods of time with a mean duration of 9–12 months [27]. Both of these drugs alone are not curative in most eumycetoma patients, but they help in localising the disease. In vitro susceptibilities of *M. mycetomatis*, the most common eumycetoma causative organism, to amphotericin B, fluconazole, itraconazole, ketoconazole, 5-flucytosine and voriconazole were determined [28]. The organism appeared to be most susceptible to the azole group; ketoconazole, itraconazole and voriconazole, with minimum inhibitory concentrations (MICs) of 0.125, 0.064 and 0.125 µg/ml, respectively [28]. Amphotericin B appeared to be less effective than ketoconazole, itraconazole and voriconazole (MIC 2 µg/ml) [29, 30]. These susceptibility tests indicate that M. mycetomatis is extremely susceptible to the azole group – ketoconazole and itraconazole, which are currently used in the medical treatment of eumycetoma caused by this organism [29, 30]. The black compound in the *M. mycetomatis* grain is melanin produced by the organism. It was thought to protect the fungus from the host immune system and antifungal agents; a fact that was proved experimentally [29, 31]. This may explain the poor response to ketoconazole and itraconazole in clinical practice [31]. The reasons for the high dropout rate in case of treatment are multifactorial. It can be due to and to the dissatisfaction of the patient due to the high cost and the prolonged treatment duration which is commonly more than one year duration, the drug side effects and complications, low socio-economic status of the patient and the lack of health education. Therefore, early diagnosis and prompt treatment is required. The long treatment duration, poor therapy response and high rate of relapse have prompted trials of novel antifungals like posaconazole, voriconazole and terbinafine. But access to drug therapies in the mycetoma belt countries remains limited due to poor availability and high cost.

Emmanuel *et al*. concluded the gruesome complications due to delay in visiting the health facility and initiation of appropriate choice of regimen [32]. Abbas *et al*., evaluated the disabling consequences of mycetoma and clear areas for intervention and further research were assessed [33]. Inclusion of mycetoma in 2016 to WHO's official list of neglected tropical disease, is a crucial step for national and global responses for addressing mycetoma, although strategic control and preventive measures are yet to be outlined [32].

In conclusion, mycetoma is a serious medical and health problem, and is associated with serious complications, low cure rate and high follow-up dropout rate. The route of infection, susceptibility and resistance in mycetoma remains poorly understood. Its low reporting and lack of familiarity may predispose patients from misdiagnosis and consequently delayed treatment. Furthermore, this is compounded by the lack of preventive and control measures. Hence health education and awareness campaign on the national and international level in the mycetoma belt is crucial. All the more, improvement in the existing and the newer modalities for early diagnosis and management in the population at risk is warranted to improve to reduce the disease morbidity and mortality.

## References

[1] Fahal A (2015) Mycetoma in the Sudan: an update from the Mycetoma Research Centre, University of Khartoum, Sudan. PLoS Neglected Tropical Diseases 9, e0003679. doi: 10.1371/journal.pntd.0003679. 25816316 PMC4376889

[2] Zijlstra EE (2016) Mycetoma: a unique neglected tropical disease. Lancet Infectious Diseases 16, 100–112. 26738840 10.1016/S1473-3099(15)00359-X

[3] Desnos-Ollivier M (2006) Molecular identification of black-grain mycetoma agents. Journal of Clinical Microbiology 44, 3517–3523. 17021076 10.1128/JCM.00862-06 PMC1594755

[4] Singh H (1979) Mycetoma in India. Indian Journal of Surgery 41, 577–597.

[5] Mathur DR (1979) An etiological and pathological study of mycetoma in Western Rajasthan. Current Medicine Research and Practice 23, 151–161.

[6] Rippon JW Medical Mycology: The Pathogenic Fungi and the Pathogenic Actinomycetes. Philadelphia: WB Saunders Co., pp. 1–797.

[7] Mattioni S (2013) Management of mycetomas in France. Medecine et Maladies Infectieuses 43, 286–294. 23916308 10.1016/j.medmal.2013.06.004

[8] Desai SC (1970) Clinical, mycological, histological and radiological studies on 40 cases of mycetomas with a note on its history and epidemiology in India. Indian Journal of Surgery 32, 427–444.

[9] Reddy CRRM (1972) Mycetoma – histological diagnosis of causal agents in 50 cases. Indian Journal of Medical Sciences 26, 733–736. 4676063

[10] Fahal A (2014) A new model for management of mycetoma in the Sudan. PLoS Neglected Tropical Diseases 8, e3271. 25356640 10.1371/journal.pntd.0003271 PMC4214669

[11] WHO (2017) Official list of Neglected Tropical Diseases. Available at https://www.who.int/neglected_diseases/diseases/en/ (Accessed 10 January 2019).

[12] Mencarini J (2016) Madura foot in Europe: diagnosis of an autochthonous case by molecular approach and review of literature. New Microbiology 39, 156–159. 27196558

[13] Welsh O (2007) Mycetoma. American Journal of Clinical Dermatology 25, 195–202.

[14] van de Sande WW (2014) Merits and pitfalls of currently used diagnostic tools in mycetoma. PLoS Neglected Tropical Diseases 8, e2918. 24992636 10.1371/journal.pntd.0002918 PMC4080999

[15] Zijlstra EE (2016) Mycetoma. Lancet Infectious Diseases 16, 100–112. 26738840 10.1016/S1473-3099(15)00359-X

[16] Fahal AH (2004) Mycetoma thorn on the flesh. Transactions of the Royal Society of Tropical Medicine and Hygiene 98, 3–11. 14702833 10.1016/s0035-9203(03)00009-9

[17] Fahal AH and Hassan MA (1992) Mycetoma. British Journal of Surgery 79, 1138–1141. 1467883 10.1002/bjs.1800791107

[18] Zein HAM (2012) The predictors of cure, amputation & follow-up dropout among mycetoma patients as seen at the Mycetoma Research Centre, University of Khartoum. Transactions of the Royal Society of Tropical Medicine and Hygiene 106, 639–644. 22854685 10.1016/j.trstmh.2012.07.003

[19] Castro LGM (2008) Clinical and mycological findings and therapeutic outcome of 27 mycetoma patients from Sao Paulo, Brazil. International Journal of Dermatology 47, 160–163. 18211487 10.1111/j.1365-4632.2008.03447.x

[20] Venkatswami S, Sankarasubramanian A and Subramanyam S (2012) The Madura foot: looking deep. International Journal of Low Extremity Wounds 11, 31–42. 10.1177/1534734612438549 22334597

[21] Sampaio FM (2013) Eumycetoma by Madurella mycetomatis with 30 years of evolution: therapeutic challenge. Anais Brasileiros de Dermatologia 88, 82–84. 24346887 10.1590/abd1806-4841.20132136 PMC3875995

[22] Bakshi R and Mathur DR (2008) Incidence and changing pattern of mycetoma in Western Rajasthan. Indian Journal of Pathology and Microbiology 51, 154–155. 18417892 10.4103/0377-4929.40433

[23] Maiti PK (1998) Mycetomas in exposed and nonexposed parts of the body: a study of 212 cases. Indian Journal of Medical Microbiology 16, 19–22.

[24] Dieng MT (2003) Mycetoma: 130 cases. Annals De Dermatologie Et De Venereologie 130, 16–19. 12605151

[25] Bonifaz A (2014) Mycetoma: experience of 482 cases in a single center in Mexico. PLoS Neglected Tropical Disease 8, 31. 10.1371/journal.pntd.0003102 PMC4140667 25144462

[26] Hassan MA and Fahal AH. (2004) Mycetoma In Kamil R and Lumby J (eds), Tropical Surgery. London, UK: Westminster Publications, pp. 786–790.

[27] Lupi O, Tyring SK and McGinnis MR (2005) Tropical dermatology: fungal tropical diseases. Journal of the American Academy of Dermatology 53, 931–951. 16310053 10.1016/j.jaad.2004.10.883

[28] Fahal AH. (2006) Mycetoma: Clinico–Pathological Monograph. Khartoum, Sudan: University of Khartoum Press, vol. 10, pp. 59–70.

[29] Ahmed AO (2004) In vitro susceptibilities of Madurella mycetomatis to itraconazole and amphotericin B assessed by a modified NCCLS method and a viability-based 2,3-Bis(2-methoxy-4-nitro-5-sulfophenyl)-5-cotrimoxazole(phenylamino)carbonyl-2H-tetrazolium hydroxide (XTT) assay. Antimicrobial Agents Chemotherapy 48, 2742–2746. 15215141 10.1128/AAC.48.7.2742-2746.2004 PMC434196

[30] van de Sande WW (2007) In vitro susceptibility of Madurella mycetomatis, prime agent of Madura foot, to tea tree oil and artemisinin. Journal of Antimicrobial Chemotherapy 59, 553–555. 17324961 10.1093/jac/dkl526

[31] van de Sande WW (2007) Melanin biosynthesis in Madurella mycetomatis and its effect on susceptibility to itraconazole and ketoconazole. Microbes and Infection 9, 1114–1123. 17644456 10.1016/j.micinf.2007.05.015

[32] Emmanuel P (2018) Mycetoma: a clinical dilemma in resource limited settings. Annals of Clinical Microbiology and Antimicrobials 17, 35. 30097030 10.1186/s12941-018-0287-4 PMC6085652

[33] Abbas M (2018) The disabling consequences of Mycetoma. PLoS Neglected Tropical Disease 12, e0007019. 10.1371/journal.pntd.0007019 PMC6312340 30532253

